# Epidemiology of appendicitis and appendectomy for the low-income population in Taiwan, 2003–2011

**DOI:** 10.1186/s12876-015-0242-1

**Published:** 2015-02-13

**Authors:** Kai-Biao Lin, Chien-Lung Chan, Nan-Ping Yang, Robert K Lai, Yuan-Hung Liu, Shun-Zhi Zhu, Ren-Hao Pan

**Affiliations:** 1 School of Computer & Information Engineering, Xiamen University of Technology, Xiamen, 361024 China; 2 Department of Information Management, Yuan Ze University, Taoyuan, 32003 Taiwan; 3 Management Center, Keelung Hospital, Ministry of Health and Welfare, Keelung City, Taiwan; 4 Institute of Public Health, National Yang-Ming University, Taipei, Taiwan; 5 Department of Computer Science and Engineering, Yuan Ze University, Taoyuan, 32003 Taiwan; 6 Innovation Center for Big data and Digital Convergence, Yuan Ze University, Taoyuan, 32003 Taiwan; 7 Section of Cardiology, Cardiovascular Center, Far Eastern Memorial Hospital, New Taipei City, Taiwan

**Keywords:** Appendicitis, Appendectomy, Epidemiology, Low-income population, Socioeconomic status

## Abstract

**Background:**

Although numerous epidemiological studies on appendicitis have been conducted worldwide, only a few studies have paid attention to the effect of socioeconomic status on appendicitis, particularly studies focusing on the low-income population (LIP).

**Methods:**

We analyzed the epidemiological features of appendicitis in Taiwan using data from the National Health Insurance Research Database from 2003 to 2011. All cases diagnosed as appendicitis were enrolled.

**Results:**

Between 2003 and 2011, 2,916 patients from the LIP and 209,206 patients from the normal population (NP) were diagnosed with appendicitis. Our finding revealed that the ratios of comorbidities, complicated appendicitis, and readmissions in LIP patients were slightly higher than those of NP patients. LIP patients were more likely to live in suburban and rural areas, and hence a higher proportion of them were hospitalized in a district or regional hospital compared with NP patients. The crucially finding was that the overall incidence ratios of appendicitis, acute appendicitis, and perforated appendicitis in the LIP were substantially higher than those in the NP (36.25%, 35.33%, and 37.28%, respectively). The mean LOS in LIP patients was longer than that of NP patients. The overall case-fatality ratio of appendectomy in the LIP was higher when compared with the NP (0.41% versus 0.12%, *p* < 0.05). We also observed that appendicitis was occurred frequently in male patients, with a higher incidence for those aged 15–29 years in both the LIP and NP. The incidences of incidental appendectomy showed a decreasing trend in both the LIP and NP. Finally, a valuable discovery was that the total hospital cost was comparable between the laparoscopic appendectomy (LA) and open appendectomy (OA) (1,178 ± 13 USD versus 1,191 ± 19 USD, *p* < 0.05) in LIP patients because they saved more hospitalization costs than NP patients when the previous one chose the LA.

**Conclusion:**

This study confirmed that a lower socioeconomic status has significantly negative impact on the occurrence and treatment of appendicitis and appendectomy. In terms of hospital costs and LOS, LIP patients benefit more from the LA approach than they do from the OA approach in the treatment of appendicitis.

## Background

Poverty is a widespread social phenomenon that not only exists in developing areas but also in developed regions [1]. Although social welfare expenditures expand continuously with changes to the social environment, poverty still exists. Demands for governmental social assistance and related welfare benefits were significantly stronger in the low-income population (LIP) [2]. The LIP is more subject to seriously disease than is the normal population (NP) in Taiwan [2-4]. Thus, it is necessary to conduct in-depth research and analyses to understand the disease condition and the cause, which can lead to suggestions for medical research institutions and governments. Appendectomy is one of the most common procedures worldwide [5], and both rich and poor patients are likely to undergo it. Therefore, for this paper, we adopted appendicitis and appendectomy as the entry point to analyze the disease conditions in LIP patients.

Although numerous epidemiological studies on appendicitis have been conducted worldwide [6-13], only a few have focused on the effect of socioeconomic status (SES) on appendicitis and appendectomy [14-17]. Studies conducted specifically on the LIP are rare. Certain studies have also been conducted in Taiwan regarding the epidemiological features of appendicitis [18-25]. These studies concerned chiefly the monthly incidence variations of acute appendicitis [19], the volume-outcome relation of acute appendicitis [20], trend differentials in incidence rates for ruptured appendicitis between rural and urban populations [21], and a comparison of the perforation rate of acute appendicitis between nationals and immigrants [22]. However, epidemiological data on appendicitis specifically for the LIP have yet to be reported in Taiwan.

This study investigates the epidemiological features, particularly age and gender, length of hospital stay (LOS), hospital cost, incidences, and seasonal variations for the LIP in Taiwan. We compared these data with the epidemiological features of the NP to determine the effect of SES on appendicitis and appendectomy. We retrieved all of the data from the National Health Insurance Research Database (NHIRD) for 2003–2011.

## Methods

### Data source

Taiwan launched a single-payer National Health Insurance (NHI) program in 1995, and its coverage rate has expanded to provide for more than 98% of the Taiwanese population since 2004. All eligible enrollees can access health care services from most clinics and hospitals by making a small copayment [26]. The National Health Insurance Bureau (NHIB) established a nationwide research database, which includes nationwide population-based data with good quality control and representation. The NHI database contains registration files and original claims data, including patient demographics, diagnosis, treatment details related to in-hospital and outpatient claims for reimbursement, and access to the NHIRD. Every claimant of the NHI program for 2003–2011 was included in the study population. Regarding the cohort study population, we traced these patients’ registration and claims data collected by the NHI program, and established the categories of expenditure according to the inpatient expenditure by admission (DD files).

For evaluating temporal trends, this study used Taiwan resident population estimates for 2003–2011 to calculate the annual rates of appendicitis and appendectomy. For all other analyses, we determined the mean annual incidence for 2003–2011 by combining the discharge data for these years, and by using the Taiwan census data as the denominator, which was created and is maintained by the Taiwan Department of Household Registration of the Ministry of the Interior. Table 1 shows the characteristics of the sampling population for the LIP based on Taiwan’s nationwide insurance dataset in 2011. On the basis of the described inclusion criteria, we included all cases identified as appendicitis, acute appendicitis, perforated appendicitis, primary appendectomy, and incident appendectomy according to the ICD9-CM code in the inpatient dataset (DD files).

**Table 1:** **Basic characteristics of the sample population for the low-income population from Taiwan’s National Health Insurance database, 2011**

Age (years)	Male		Female		All	
	n	%	n	%	n	%
0-14 y/o	45,821	29.21%	43,927	28.64%	89,748	28.93%
15-29 y/o	35,571	22.67%	43,419	28.31%	78,990	25.46%
30-44 y/o	20,154	12.85%	33,832	22.06%	53,986	17.40%
45-59 y/o	33,723	21.50%	22,228	14.49%	55,951	18.03%
60-74 y/o	15,133	9.65%	6,045	3.94%	21,178	6.83%
75 y/o or more	6,482	4.13%	3,938	2.57%	10,420	3.36%
Total	156,884	50.56%	153,389	49.44%	310,273	100.00%

### Data protection and permission

The personal information of all subjects was encrypted with a double scrambling protocol for research purposes to protect the privacy of the patients. All researchers who wish to use the NHIRD and its data subsets are required to sign a written agreement declaring that they have no intention of attempting to obtain information that could potentially violate the privacy of patients or care providers. This study was approved by the Institutional Review Board (IRB) of Taoyuan General Hospital, which has been certified by the Ministry of Health & Welfare, Taiwan (IRB Approval Number: TYGH103015), and the protocol was evaluated by the National Health Research Institutes (NHRI), which consented to this planned analysis of the NHIRD (Agreement Number: NHIRD-103-160).

### Data definition

To investigate the incidence of appendicitis in Taiwan, we used the International Classification of Diseases, Ninth Revision, Clinical Modification (ICD-9-CM) diagnosis codes in this study. Appendicitis comprised the diagnosis codes of 540 (acute appendicitis), 541 (appendicitis, unqualified), 542 (other appendicitis), and 543 (other disease of the appendix). Acute appendicitis refers to only the diagnosis code of 540 (acute appendicitis), which is further classified as 540.0 (acute appendicitis, with generalized peritonitis), 540.1 (acute appendicitis, with peritoneal abscess), and 540.9 (acute appendicitis, without mention of peritonitis). The terms appendicitis and acute appendicitis are not interchangeable in this paper. Appendicitis not only refers to acute appendicitis (540), but also includes the diagnosis code of 541, 542 and 543. However, when we mention acute appendicitis, we use the term acute appendicitis explicitly and consistently. The procedure codes were 47.0 (appendectomy, excludes incidental) and 47.1 (incidental appendectomy). Perforated appendicitis was considered present with appendectomies showing evidence of perforation, peritonitis, rupture, or abscess (ICD-9-CM diagnostic codes 540.0 and 540.1). The perforation ratio was defined as the ratio of the number of perforated appendectomies to the number of appendectomies. Diagnostic accuracy was defined as the proportion of all appendectomy patients who were provided a discharge diagnosis of appendicitis [27-29]. This concept is equivalent to the positive predictive value of the surgeon's preoperative diagnosis, leading to an appendectomy. Comorbidities were identified by referring to the ICD-9-CM codes, as described in Appendix C in [23]. Complicated appendicitis was defined as appendicitis with perforation, abscess formation, or peritonitis. Readmission for complication was defined as readmission with the diagnosis of a commonly encountered postoperative complication within 1 month after an appendectomy (Appendix B in [23]). The case-fatality ratio was defined as the percentage of patients with appendectomy who died during hospitalization.

### Classification of LIP and NP

To evaluate the socioeconomic effect, the enrolled subjects were divided into NP and LIP groups if they satisfied the criteria of Taiwan’s Social Assistance Act, and were registered in Taiwan’s NHI database. Low-income households were defined as those with a monthly average per-member gross income of less than the monthly minimum living expense standard of that residence region. The minimum living expense standard was defined as 60% of the average monthly disposable income for each region. The family property must not exceed a certain amount, as determined by the central or municipal authorities in the corresponding year [30]. This subpopulation was recorded as the fifth class insured in Taiwan’s NHI database [26]. The NP refers to those who are not part of the LIP; that is, the subpopulation of the total population excluding the LIP.

### Outcome of measurement

#### Length of hospital stay

The period between admission and discharge was defined as the LOS (measured in days). The LOS was recorded as 1 day for patients discharged on the same day they were admitted to the hospital [23].

#### Hospital costs

The hospital costs were calculated by summing all the items enumerated in the hospital discharge summary, including operation-associated costs and ward costs. The operation-associated costs included anesthesia and surgery fees as well as costs of medical supplies used during the operation. The surplus costs were classified as ward costs. The costs expressed in this study are in U.S. dollars (USD). In 2007, 1 USD dollar was equivalent to approximately 32.64 Taiwan dollars [23].

### Statistical analysis

For analysis, descriptive statistics for a comparison of the baseline characteristics were represented by the number of cases, percentages, annual incidence rates (per 100,000 people), and 95% confidence intervals (CI) for the estimated rates. Pearson’s chi-square (χ2) was used to evaluate the statistical significance difference of non-continuous variables between LIP and NP, and the Analysis of Variance (ANOVA) was used to describe and compare continuous variables among different subgroups. The significance level was set at *p =* 0.05. To evaluate the risk factors of perforated appendicitis, multiple logistic regression method was used and the Odds Ratio (OR) was calculated. To estimate the incidence of different populations in each age group, we constructed a life table in 15-year age intervals by using combined incidence data from 2003 to 2011. To compare the incidence of appendicitis in different months and seasons, we adjusted months with fewer than 31 days to fit a standard month of 31 days. To reduce the impact of extreme data on the mean of LOS and hospital costs, we excluded 1% of maximum values and 1% of minimum values from the raw data. All statistical analyses were performed using the Statistical Package for Social Sciences for Windows (SPSS for Windows Version 18.0).

## Results

During 2003–2011, 2,916 patients from the LIP and 209,206 patients from the NP were diagnosed with appendicitis. As shown in Table 2, 4.79% of LIP patients had one comorbidity, 0.52% had more than one comorbidity; 26.54% of LIP patients underwent complicated appendicitis, and 3.29% of LIP patients were readmitted to hospital because of complications. All the demographic characteristics for LIP patients were slightly higher compared with NP patients. In addition, compared with the NP patients, LIP patients were more likely to live in suburban (22.15% versus 13.24%) and rural areas (1.88% versus 0.98%), and a higher proportion of them were hospitalized in a district or regional hospital (23.39% versus 16.55% and 52.17% versus 49.21%, respectively). The overall case-fatality ratio for appendectomy was higher compared with the NP group (0.41% versus 0.12%, *p* < 0.05), and the diagnostic accuracy was similar between LIP and NP groups (97.06% versus 96.37%, respectively, *p* < 0.05).

**Table 2:** **Demographic characteristics for low-income population and normal population patients with appendicitis in Taiwan between 2003 and 2011**

Variable	Low income population (n = 2,916)		Normal population (n = 209,206)		*p*value
	n	%	n	%	
**Gender** ^**a**^					0.000
Female	1,455	49.90%	112,421	53.74%	
Male	1,455	49.90%	95,551	45.67%	
Undefined	6	0.21%	1,234	0.59%	
**Comorbidities** ^**c**^					0.000
0	2,766	94.69%	198,878	94.93%	
1	140	4.79%	9,714	4.64%	
≥2	15	0.52%	912	0.43%	
**Complicated appendicitis**					0.000
No	2,142	73.46%	154,798	73.99%	
Yes	774	26.54%	54,408	26.01%	
**Readmission for complication**					0.003
No	2,820	96.71%	202,830	96.95%	
Yes	96	3.29%	6,376	3.05%	
**Hospital mortality**	0.000				
No	2,904	99.59%	208,953	99.88%	
Yes	12	0.41%	253	0.12%	
**Diagnostic accuracy** ^**b**^					0.004
No	79	2.94%	7,162	3.63%	
Yes	2,608	97.06%	190,166	96.37%	
**Hospital level** ^**c**^					0.000
District hospital	686	23.39%	34,876	16.55%	
Regional hospital	1,530	52.17%	103,730	49.21%	
Medical center	717	24.45%	72,175	34.24%	
**Area level** ^**c**^					0.000
Urban	2,219	75.97%	179,919	85.78%	
Suburban	647	22.15%	27,761	13.24%	
Rural	55	1.88%	2,064	0.98%	

^a^“Undefined” indicates that information regarding sex is missing.

^b^The denominator of “Diagnostic Accuracy” was the total number of patients with primary appendectomy (2,687 LIP patients and 197,328 NP patients).

^c^The sum number of hospital level, area level, and comorbidities was larger than the total patients in each subgroups, because some patients with appendicitis may be admitted to different hospital (the hospitals may differ in hospital level or area level), or with different comorbidities at different times.

### Appendicitis

**Figure 1:**
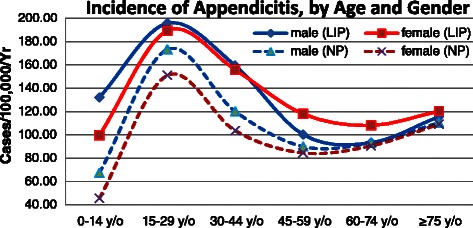
**Annual incidence of appendicitis (per 100,000 people) in Taiwan according to socioeconomic status, age group, and sex, 2003–2011.**

The overall incidence of appendicitis was 139.54 per 100,000 per year (95% CI: 132.22-146.85) for the LIP, and 102.41 per 100,000 per year (95% CI: 96.14-108.68) for the NP (*p* < 0.05). The overall LIP-NP ratio of incidence for appendicitis was 1.36:1. The age-specific incidence of appendicitis for the LIP exhibited a similar pattern between males and females, and was highest for the age group of 15–29 years for both sexes; this phenomenon was consistent with the NP. The incidences for the LIP were higher than that for the NP in all age groups for both sexes. The greatest difference in the incidence of appendicitis was in the 0–14 years group, in which the incidence for the LIP (132.12 per 100,000 per year, 95% CI: 125.00-139.24) was 96.01% higher than that for the NP (67.40 per 100,000 per year, 95% CI: 62.32-72.49) in males (*p* < 0.05 ), and the incidence for the LIP (99.56 per 100,000 per year, 95% CI: 93.38-105.74) was 118.35% higher than that of the NP (45.60 per 100,000 per year, 95% CI: 41.41-49.78) in females (*p* < 0.05) (Figure 1).

### Acute appendicitis

**Figure 2:**
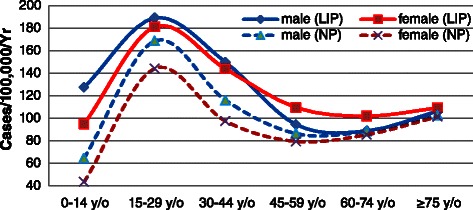
**Annual incidence of acute appendicitis (per 100,000 people) in Taiwan according to socioeconomic status, age group, and sex, 2003–2011.**

**Figure 3:**
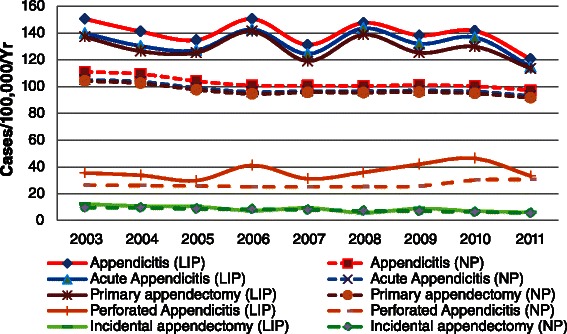
**Secular trend of incidence for appendicitis, acute appendicitis, primary appendectomy, perforated appendicitis, and incidental appendectomy in Taiwan, 2003–2011.**

In total, 2,770 LIP patients, accounting for 94.99% of all LIP appendicitis patients, and 199,813 NP patients had been diagnosed with acute appendicitis. The overall incidence of acute appendicitis was 132.37 per 100,000 per year (95% CI: 125.24-139.50) in the LIP, which was 35.33% higher than the value of 97.81 per 100,000 per year (95% CI: 91.68-103.94) in the NP (*p* < 0.05). The age-specific incidence of acute appendicitis and appendicitis exhibited a similar pattern (Figure 1 versus Figure 2), and the secular trend followed the same pattern as well (Figure 3).

### Primary appendectomy

**Figure 4:**
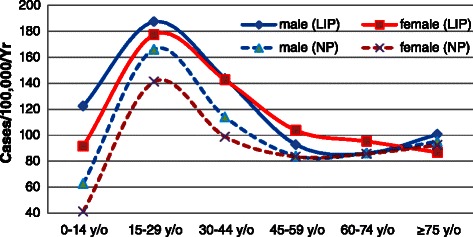
**Annual incidence of appendectomy (per 100,000 people) in Taiwan according to socioeconomic status, age group, and sex, 2003–2011.**

A primary appendectomy was defined as a non-incidental appendectomy. For LIP patients, a total of 2,687 patients underwent a primary appendectomy. Among them, 2,533 patients (94.27%) were diagnosed with acute appendicitis, 75 patients (2.79%) were diagnosed with ICD codes of 541–543 (unqualified appendicitis, other appendicitis or other disease of the appendix), and the remaining 79 patients (2.94%) were recorded without the diagnosis of appendicitis. For NP patients, a total of 197,328 patients underwent a primary appendectomy. Among them, 184,499 patients (93.50%) were diagnosed with acute appendicitis, 5,667 patients (2.87%) were diagnosed with ICD codes of 541–543, and the remaining 7,162 patients (3.63%) were recorded without the diagnosis of appendicitis. The overall incidence of primary appendectomy was 128.47 per 100,000 per year (95% CI: 121.45-135.49) in the LIP. The age-specific incidence of primary appendectomy exhibited a similar pattern for both sexes, and those at greatest risk were LIP patients aged 15–29 years. The overall incidence for the LIP was 33.0% higher than that for the NP (96.59 per 100,000 per year, 95% CI: 90.51-102.68) (*p* < 0.05). For males, the incidence in the LIP was higher for all ages compared with the NP; the greatest difference emerged in the age group of 0–14 years, in which the incidence in the LIP was 94.81% higher than that in the NP (*p* < 0.05). Female patients were primary in the same situation as their male counterparts, with the main difference being that the incidence in the LIP was 6.42% lower than that in the NP for the age group of 75 years and older (*p* < 0.05), showing that this phenomenon was rare (Figure 4).

### Incidental appendectomy

**Figure 5:**
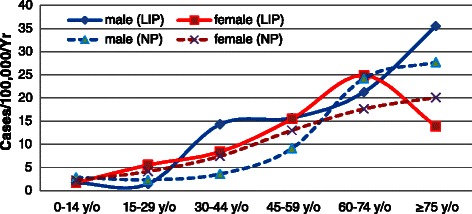
**Annual incidence of incidental appendectomy (per 100,000 people) in Taiwan according to socioeconomic status, age group, and sex, 2003–2011.**

In total, 178 LIP patients and 15,926 NP patients underwent an incidental appendectomy. The overall incidence of incidental appendectomy was 8.69 per 100,000 per year (95% CI: 6.87-10.52) in the LIP, which was 11.4% higher than that in the NP (7.80 per 100,000 per year; 95% CI: 6.07-9.54) (*p* > 0.05). The overall incidence of incidental appendectomy was higher for males than for females in the LIP, with an overall male–female ratio of 1.29:1. This situation was reversed in the NP, which had an overall male–female ratio of 0.86:1. The median age of incidental appendectomy patients was 52(40, 70) years for LIP patients and 54(41, 68) years for NP patients. The annual incidence of incidental appendectomy gradually increased with age in both the LIP and the NP for almost all ages, except for females aged 75 years or older in the LIP, whose rate was lower than that of the age group of 45–74 years (Figure 5).

### Perforated appendicitis

In total, 774 LIP patients and 54,408 NP patients were diagnosed with appendiceal perforation, rupture, abscess, or generalized peritonitis. Among LIP patients, 57.62% were male, and 42.38% were female. The overall incidence of perforated appendicitis was 36.54 per 100,000 per year (95% CI: 32.80-40.29) in the LIP, and 26.62 per 100,000 per year (95% CI: 23.42-29.82) in the NP (*p* < 0.05), with an overall LIP-NP incidence ratio of 1.37:1. As shown in Table 3, among all appendicitis cases, the male, the younger (aged 14 years or younger), and the 30 years or older groups had higher risk of perforated appendicitis, and the rate of ruptured appendicitis increased with age among adults. We also observed an increased risk of perforation if patients had one or more comorbidity, and the risk increased as the number of comorbidities grew. Furthermore, patients admitted to regional hospitals and medical centers had a higher risk of perforation than those admitted to district hospitals had, and patients admitted to medical centers had a higher risk than those admitted to regional hospitals had. This phenomenon was consistent in both NP and LIP patients. No statistically significant difference was found in the variable of comorbidities (*p* = 0.126 if number of comorbidities was one, and p = 0.218 if number of comorbidities was 2 or larger) in LIP patients (Table 3).

**Table 3:** **Multiple logistic regression analysis of the risk factors for perforation in LIP patients and NP patients with appendicitis in Taiwan, 2003-2011**

Variable	low-income population		Normal population	
	AOR (95% CI)	*p*	AOR (95% CI)	*p*
**Gender**				
Female	1		1	
Male	1.40 (1.18,1.67)	<0.001	1.34 (1.31,1.36)	<0.001
**Age (years)**				
0-14 y/o	1.98 (1.56,2.50)	<0.001	2.22 (2.15,2.30)	<0.001
15-29 y/o	1		1	
30-44 y/o	1.62 (1.25,2.09)	<0.001	1.36 (1.32,1.40)	<0.001
45-59 y/o	2.78 (2.11,3.67)	<0.001	2.22 (2.15,2.29)	<0.001
≥60 y/o	4.44 (3.27,6.05)	<0.001	3.74 (3.62,3.87)	<0.001
**Comorbidities**				
0	1		1	
1	1.34 (0.92,1.95)	0.126	1.46 (1.40,1.53)	<0.001
≥2	1.96 (0.67,5.68)	0.218	1.67 (1.47,1.91)	<0.001
**Hospital Level**				
District hospital	1		1	
Regional hospital	1.39 (1.11,1.73)	<0.001	1.33 (1.29,1.37)	<0.001
Medical center	1.95 (1.53,2.50)	<0.001	1.52 (1.47,1.57)	<0.001

AOR: adjusted odds ratio.

Multiple linear regression was conducted after adjustment for age, gender, comorbidities, and hospital level, but not the target variable.

**Figure 6:**
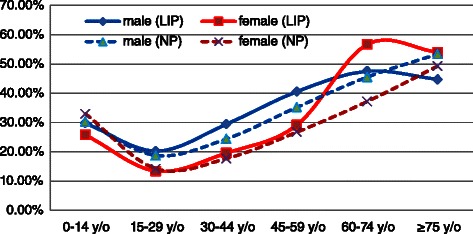
**Perforation ratios (per 100,000 people) in Taiwan according to socioeconomic status, age group, and sex, 2003–2011.**

The overall perforation ratio was 26.54% in the LIP patients, which was similar to the value of 26.01% in the NP patients. For males, this value was 30.65% in the LIP, which was slightly higher than the value in the NP (28.29%). For females, the values were 22.54% in the LIP, and 23.41% in the NP. Age-specific perforation ratios were similar for both sexes in the LIP; they were lowest in the 15–29 years old group and highest in the 60–74 years old group, and the ratios increased gradually with age for all ages, excluding the age groups of 0–14 years and 75 years or older (Figure 6).

### Utilization of care (LOS and Hospital Cost)

From 2003 to 2011, LIP patients with appendicitis accounted for an estimated 15,499 hospital days (1,722 per year) in total, and 5.34 ± 0.09 days on average per person, which was 13.13% higher than the 4.72 ± 0.01 days per NP patient (*p* < 0.05). Moreover, in the LIP, the mean LOS was 5.15 ± 0.09 days for patients with acute appendicitis, 5.15 ± 0.09 days for patients with performed appendectomy, 8.57 ± 0.23 days for patients with a perforated appendix, and 14.74 ± 0.73 days for patients with incidental appendectomy performed during another surgical procedure. As shown in Table 4, the LOS for all appendicitis types and appendectomy types among LIP patients was longer than that among NP patients. For LIP patients, the greatest difference concerned perforated appendicitis, with an LIP-NP ratio of 1.15:1. The average hospital costs of appendicitis, acute appendicitis and perforated appendicitis were higher in the LIP patients compare with the NP patients (LIP-NP ratio: 1.06, 1.05 and 1.10, respectively). However, the average costs of primary appendectomy and incidental appendectomy were similar (LIP-NP ratio: 1.01 and 0.99, respectively) (Table 4).

**Table 4:** **The LOS and hospital cost for all appendicitis types and appendectomy types between LIP patients and NP patients**

Variable	Socioeconomic status	Appendicitis	Acute appendicitis	Primary appendectomy	Perforated appendicitis	Incidental appendectomy
Mean hospital stay ± SE (days)	LIP	5.34 ± 0.09	5.15 ± 0.09	5.15 ± 0.09	8.57 ± 0.23	14.74 ± 0.73
	NP	4.72 ± 0.01	4.64 ± 0.01	4.78 ± 0.01	7.46 ± 0.02	13.75 ± 0.07
	LIP-NP ratio	1.13	1.11	1.08	1.15	1.07
Mean hospital cost ± SE (US$)	LIP	1,157 ± 14	1,133 ± 13	1,188 ± 15	1,679 ± 43	3,561 ± 178
	NP	1,093 ± 1	1,081 ± 1	1,173 ± 2	1,523 ± 5	3,612 ± 19
	LIP-NP ratio	1.06	1.05	1.01	1.10	0.99

SE: standard error of the mean.

To reduce the impact of extreme data on the mean of LOS and hospital cost, we excluded 1% of maximum values and 1% of minimum values from the raw data.

Table 5 shows the comparison of the medical utilization between the operation type of open appendectomy (OA) and laparoscopic appendectomy (LA), revealing that more patients undertook OA than LA in both LIP and NP patients (77.30% versus 22.70% in LIP patients, and 72.80% versus 27.52% in NP patients, respectively), and LIP patients were less likely to select a LA compared with NP patients (22.70% versus 27.52%, *p* < 0.05). As shown in Table 5, LA was correlated with a significantly shorter LOS compared with OA (3.80 ± 0.08 versus 5.51 ± 0.11 for LIP patients, and 3.80 ± 0.01 versus 5.17 ± 0.01 for NP patients, respectively). The mean LOS of OA for LIP was longer compared with NP (5.51 ± 0.11 versus 5.17 ± 0.01, *p* < 0.05), but the values were similar for LA in both LIP and NP (3.80 ± 0.08 versus 3.80 ± 0.01, *p* < 0.05). For NP, the average cost for LA was slightly higher than that for OA (1,180 ± 1 versus 1,170 ± 2 USD, *p* < 0.05). However, the average cost for LA was slightly less than that for OA for NP (1,178 ± 13 versus 1,191 ± 19 USD, *p* < 0.05) (Table 5).

**Table 5:** **Medical utilization of appendectomy in Taiwan by socioeconomic status and operation type, 2003-2011**

Socioeconomic status	Operation type	Summed cases 2003-2011 (%)	LOS (days) Mean (SE)	Cost (USD) Mean (SE)
**LIP**	OA	77.30%	5.51 ± 0.11	1,191 ± 19
	LA	22.70%	3.80 ± 0.08	1,178 ± 13
ANOVA test			*p = 0.000*	*p = 0.000*
**NP**	OA	72.48%	5.17 ± 0.01	1,170 ± 2
	LA	27.52%	3.80 ± 0.01	1,180 ± 1
ANOVA test			*p = 0.000*	*p = 0.000*

OA: Open appendectomy LA: Laparoscopic appendectomy.

To reduce the impact of extreme data on the mean of LOS and hospital cost, we excluded 1% of maximum values and 1% of minimum values from the raw data.

### Seasonal variation

**Figure 7:**
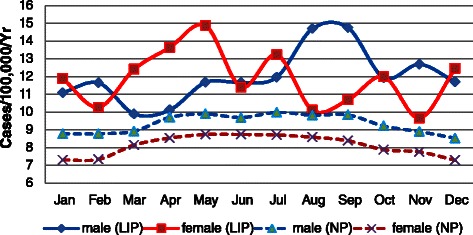
**Monthly incidence of appendicitis in Taiwan, 2003–2011.** Adjusted to 31-day monthly totals, annual data combined.

The incidence of appendicitis revealed a clear seasonality for males and females in the NP, peaking during the summer months with a slump during the winter months. We also observed a slightly higher incidence in summer (36.55 per 100,000 per season, 95% CI: 32.80-40.30) than in winter (34.60 per 100,000 per season, 95% CI: 30.95-38.25) from the overall LIP data (*p* > 0.05). It is difficult to determine an obvious seasonality in the LIP, but it is easy to determine changes in monthly incidence in opposite directions between males and females. The incidence was higher in the LIP than in the NP in every month for both sexes (Figure 7).

### Secular trends, 2003–2011

From 2003 to 2011, the overall annual incidence of appendicitis in the LIP did not reveal a clear trend, and mostly assumed a wavy shape from year to year. However, the overall annual incidence of appendicitis in the NP revealed a clear and steady downward trend. The annual incidence of appendicitis in the LIP was substantially higher than in the NP for every year. A similar pattern of a secular trend also emerged in the annual incidence of acute appendicitis and primary appendectomy, presenting an irregular annual incidence in the LIP. The overall incidence of perforated appendicitis appeared as a slightly upward trend in the LIP, but it is difficult to identify the regularity, which occurred with a high incidence in certain years, but with a lower incidence in other years. The incidences of perforated appendicitis in the LIP were higher than in the NP for every year. The annual incidence of incidental appendectomy in the LIP exhibited a gradually declining trend, and this was consistent with that in the NP (Figure 3).

## Discussion

According to the provision of Section 13 and Paragraph 3 of the Taiwan Social Assistance Act, “The competent authority must hold living conditions survey for low-income population and moderate low income population at least every five years, and publish statistical reports” [30]. The Taiwan Ministry of the Interior had conducted six surveys regarding the living conditions in the LIP and moderate LIP. The first survey was conducted in 1990, and the latest was performed in 2013 [31]; the statistical reports from 2013 have not been presented yet. According to the survey results of 2004, the five main reasons for people becoming a part of the LIP were as follows: all family members are unable to work (35.33%), long-term illness (26.93%), many people in the family are unable to work (24.22%), other reasons (11.56%), and income earners who are divorced or separated (11.03%) [2]. Therefore, long-term illness is the second leading cause of people becoming poor in Taiwan. In other words, long-term illness led to 62,554 people becoming a part of the LIP in 2004 (the LIP comprised 232,284 people in 2004). The survey results also showed that the proportion of families who had family members with a chronic or catastrophic illness reached 62.09% in low-income families in 2004, when the total number of families in the LIP was 78,428. Among them, families with one patient accounted for 51.73% of the total number of low-income families, families with two patients was accounted for 8.57%, and families with three or more patients accounted for 1.79%. The survey results in 2008 were similar to those of 2004 [4]. For example, the proportion of families who had family members with a chronic or catastrophic illness reached 65.24% in low-income families in 2008, which was 3.15% higher than as indicated in the survey results of 2004. The situation in the LIP with disease was more serious than in the NP; hence, in-depth research and analyses is necessary for understanding the disease condition and the cause, to provide suggestions for medical research institutions and the government.

Previous studies have provided different definitions of appendicitis. For example, certain studies have defined a diagnosis of appendicitis as patients who had undergone an appendectomy [5,32,33]. David et al. [10] proposed that a patient with a positive primary appendectomy was considered to have acute appendicitis; the terms were used interchangeably in their paper. Lee et al. [6] defined appendicitis as acute appendicitis (K35), other appendicitis (K36), and unspecified appendicitis (K37) according to the ICD-10. The definition in this study was similar to that by Lee et al. [6], who applied a diagnosis of appendicitis, regardless of whether subjects underwent an appendectomy. This definition can more accurately distinguish between appendicitis, acute appendicitis, and appendectomy. However, it increases the incidence of appendicitis, causing it to be slightly higher than when the other aforementioned definitions are used. In the present study, we focused on comparing the LIP and the NP, and used the same definition for both groups; therefore, our results were not significantly affected.

The overall incidences of appendicitis, acute appendicitis, appendectomy, and perforated appendicitis in the LIP were 36.25%, 35.33%, 33.00%, and 37.28% higher than those in the NP, respectively. In other words, the risks of all aforementioned appendicitis in the LIP were higher than in the NP; therefore, an appendectomy was also more frequently performed in the LIP. To explore the reasons for the higher incidence in the LIP, we reviewed certain etiologic hypotheses on appendicitis to determine any association.

Numerous hypotheses have been proposed to explain the etiology of appendicitis, but only three of them have a measure of credibility and warrant further discussion [34]. The first etiologic hypothesis was the mechanical hypothesis, which was proposed by Short [35] in 1920. He hypothesized a causal relationship of appendicitis with a low cellulose content of imported foods. To the best of our knowledge, although the diet quality in the LIP may be less favorable than in the NP, differences in eating habits between the LIP and the NP were not substantial in Taiwan. Therefore, the possibility that the diet in the LIP contains more low-fiber foods compared with the NP was low. In addition, Barker and Liggins [36] found that, despite similar dietary habits, the distribution of appendicitis did not follow other diseases associated with low fiber consumption. Therefore, there is a small possibility of a low-fiber diet leading to the higher incidence in the LIP than in the NP, and thus, we excluded eating habits as the reason for the higher incidence of appendicitis in the LIP than in the NP. The second etiologic hypothesis was the infection hypothesis; specific infections with viruses, bacteria, and parasites have been linked to appendicitis, prompting the suggestion that a local invasion could trigger appendicitis [37-40]. We agree with the infection hypothesis because it is possible that the LIP is more likely to be infected than the NP, resulting in the higher incidence of appendicitis in the LIP than in the NP. The third hypothesis is the hygiene hypothesis. The effect of better socioeconomic conditions because of improved water supplies and hygiene conditions has been found to be a reason for the decrease in the incidence of appendicitis [41,42]. We conjectured two possible reasons for the higher incidence of appendicitis in the LIP than in the NP, as follows: the LIP is infected more easily, and the LIP is under less favorable hygiene conditions. However, these conjectures warrant further research and in-depth clinical trials for verification, which we plan to conduct in the next phase of work. Our findings also revealed that the mean LOS for LIP patients with appendicitis, acute appendicitis, primary appendectomy, perforated appendicitis, and incidental appendectomy was 13.12%, 10.94%, 7.84%, 14.79%, and 7.18% longer compared with NP patients, respectively. This may be caused by three reasons. First, LIP patients may live in a more remote area than NP patients do; they tend to be uninsured, and may need to travel farther than NP patients do to obtain medical care [21]. This may lead to a serious disease by the time they arrive at a hospital because of the delay, and hence, they may need a long LOS. This may also be the reason that a higher incidence of perforated appendicitis was found in the LIP compared with the NP. Second, poor financial conditions may result in a poor quality of life, and therefore, the constitution of the LIP may be weaker than that of the NP, thereby requiring a lengthier recovery time after an appendectomy. Finally, a certain relationship with the health care system in Taiwan may affect the incidence as well. Because LIP patients are not required to pay any fees covering hospital costs according to health care provisions in Taiwan, certain LIP patients may be less likely to consider payment problems for long LOS when they use medical resources.

Based on our experience, most appendectomy was caused by acute appendicitis, which is verified in our study (94.27% for LIP patients, 93.50% for NP patients, respectively). However, we also found that some patients who underwent appendectomy were diagnosed with ICD codes of 541–543 (unqualified appendicitis, other appendicitis or other disease of the appendix) (2.79% for LIP patients, 2.87% for NP patients, respectively). Because the surgery for chronic appendicitis is rare, this situation is an interesting phenomenon. The primary reason for this is that some patients may require appendectomy even when the symptoms for acute appendicitis are not obvious. Some physicians’ improper coding behavior may also lead to this situation, which needs further study to clarify.

LA is not routinely performed for appendicitis because the operation costs associated with that procedure are higher than those associated with OA. However, our findings revealed that the total cost was comparable between the LA and OA (1,191 ± 19 USD of OA versus 1,178 ± 13 USD of LA, *p* < 0.05) in LIP patients. The mean LOS of OA for LIP patients was longer than that for NP patients (5.51 ± 0.11 versus 5.17 ± 0.01, *p* < 0.05), but it was similar for LA between LIP and NP patients (3.80 ± 0.08 versus 3.80 ± 0.01, *p* < 0.05); hence, more hospitalization costs are saved when LIP patients chose the operation type of LA. In terms of hospital costs and LOS, LIP patients benefit more from the LA approach for the treatment of appendicitis. Nevertheless, more prospective investigations should be designed to explore the economic advantages of LA, such as the time back to work and normal daily activity [23].

The overall incidence of appendicitis, acute appendicitis, and primary appendectomy appeared to be strongly age related in both the LIP and the NP, with the highest incidence in those aged 15–29 years, but a lower incidence in the younger and older age groups. In addition, the incidence of perforated appendicitis appeared to be age related in both the LIP and the NP, and was highest in older people and lower in younger people. The perforation ratio was also strongly age related in both population groups, and was highest in older people and lowest for the age group of 15–29 years. This phenomenon has also been observed in previous studies [6,10,43,44], in which the researchers had called it “J-shaped”. Furthermore, some of these studies have divided the 0-14-years old group into three groups; they found that the perforation ratio of 0–4 years was extremely high. By referring to their classification method that divided the age group into three age groups, our data also revealed the same characteristics (the data are not presented in the paper). As David et al. [10] stated, this pattern reflects both the increased diagnostic difficulty and less timely surgical intervention for people in these extreme age groups.

Regarding seasonal variations, although the incidence of appendicitis in the LIP did not exhibit an obvious regularity as it did in the NP, we observed that the incidence was slightly higher in summer than in winter based on the overall data. This pattern has been observed in previous studies as well [6,10,19,33,45]. Wei et al. [19] analyzed the relationship between the incidence of appendicitis and climate factors, including ambient temperature, relative humidity, atmospheric pressure, rainfall, and hours of sunshine, and they found that only the ambient temperature was positively correlated with the incidence of appendicitis. Kaplan et al. [46] reported a significant effect of air pollution on the incidence of appendicitis in the summer months. Several factors may contribute to the seasonality of appendicitis and appendectomy, but no single causative factor has been identified [6,10,32].

The overall incidence of incidental appendectomy in the LIP was 8.69 per 100,000 per year, which was 11.4% higher than in the NP (7.80 per 100,000 per year); however, this is lower than what has been found in certain studies [10]. The overall incidence of incidental appendectomy decreased by 43.3% between 2003 and 2011, and the decline trend is consistent with the findings in previous studies [10], but the decline ratio was greater.

The NHIB has established a uniform system to control the quality of medical services and coding, and therefore, the quality of data acquisition in the present study was reliable [20,47,48]. However, our data are still subject to limitations. In total, 1,240 records of appendicitis patients were missing information regarding sex (six LIP patient records and 1,234 NP patient records) between the years 2003 and 2004; in one record the sex information was absent in 2006 and 2010. However, sex information for the other years was complete. The missing sex information did not affect the calculation of the overall incidence unrelated to sex information, but certain errors are to emerge when we conduct a comparison of the incidence in males and females at different ages. To resolve this problem, we calculated the number of records for male and female patients in each age group because those records contained sex information, and then we divided the number of males by that of females to obtain a male–female ratio. Afterward, we randomly assigned records of the same age group without sex information to the male or female groups according to the obtained sex ratio. This solution retained the total number of records as unchanged, and ensured that the male–female ratio was relatively accurate, but it still resulted in some deviation, which is one drawback of our study.

## Conclusions

This study revealed that the overall incidence of appendicitis, acute appendicitis, and perforated appendicitis in the LIP was substantially higher than that in the NP. The trend of incidence for the LIP did not exhibit an obvious decline trend as the NP did during the observation period. The mean LOS in the LIP patients was longer than that in the NP patients. Furthermore, the overall case-fatality ratio of appendectomy in the LIP was higher than that in the NP. On the basis of these findings, we confirmed that a lower SES has significantly negative impact on the occurrence and treatment of appendicitis and appendectomy. Another crucial finding in our study was that the total hospital cost was comparable between LA and OA in LIP patients. LIP patients benefit more from the LA approach in the treatment of appendicitis when costs and LOS were considered because LIP patients will save more hospital ward costs than NP patients did when the previous one chose LA.
